# Supplementary material for: Seasonal enhancement of the viral shunt catalyzes a subsurface oxygen maximum in the Sargasso Sea
Source: Nat Commun. 2025 Dec 6;17:352. doi: 10.1038/s41467-025-67002-1 (PMC12796325; doi:10.1038/s41467-025-67002-1)
Supplement: Supplementary file 1 — Supplementary Information [file 41467_2025_67002_MOESM1_ESM.pdf]

Supplemental Information for

Seasonal Enhancement of the Viral Shunt Catalyzes a Subsurface Oxygen Maximum in the Sargasso Sea

Author list

Naomi E. Gilbert, Daniel Muratore, Camelia Shopen Gochev, Gary R. LeCleir, Shelby M. Cagle, Helena L. Pound, Christine L. Sun, Alfonso Carrillo, Kimberley S. Ndlovu, Ilia Maidanik, Ashley R. Coenen, Lauren Chittick, Jennifer M. DeBruyn, Alison Buchan, Debbie Lindell, Matthew B. Sullivan, Joshua S. Weitz#, and Steven W. Wilhelm#

#authors for correspondence

Supplementary Figures

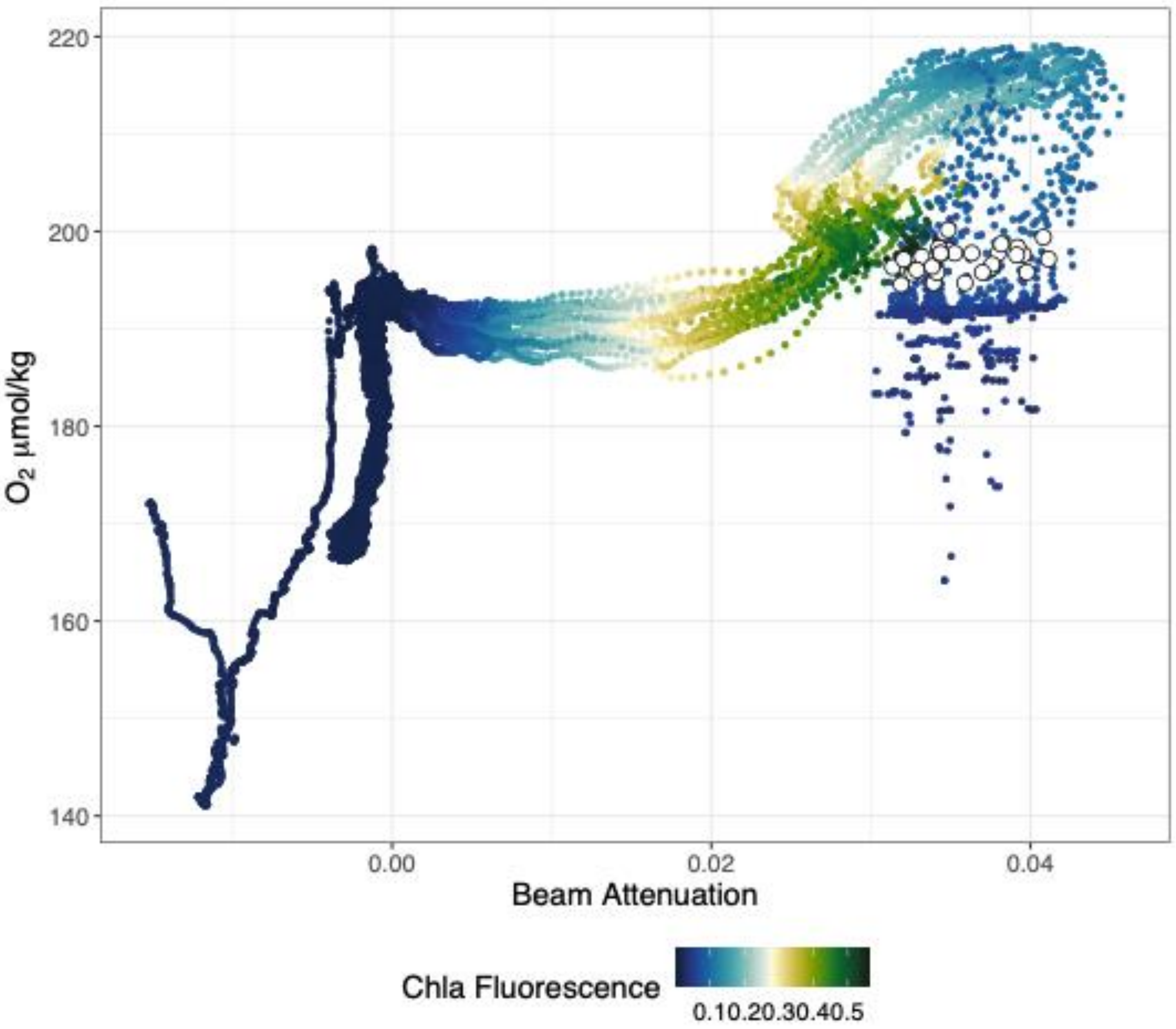

Supplementary Figure 1. CTD data relating oxygen concentration, beam attenuation, and chlorophyll fluorescence. Y-axis shows CTD-optode derived oxygen concentration, x-axis shows beam transmissometer-based beam attenuation as a proxy for particle concentrations, and point color shows chlorophyll a fluorescence as measured by CTD fluorimeter (subject to the effects of quenching). Each colored point represents a 2db binned average value from a CTD case in the depth range of 5-500m during the October 2019 Lagrangian framework cruise AE1926. Solid points indicate the mixed layer depth as determined by the 0.125 kg/m^3 potential density differential described in Methods.

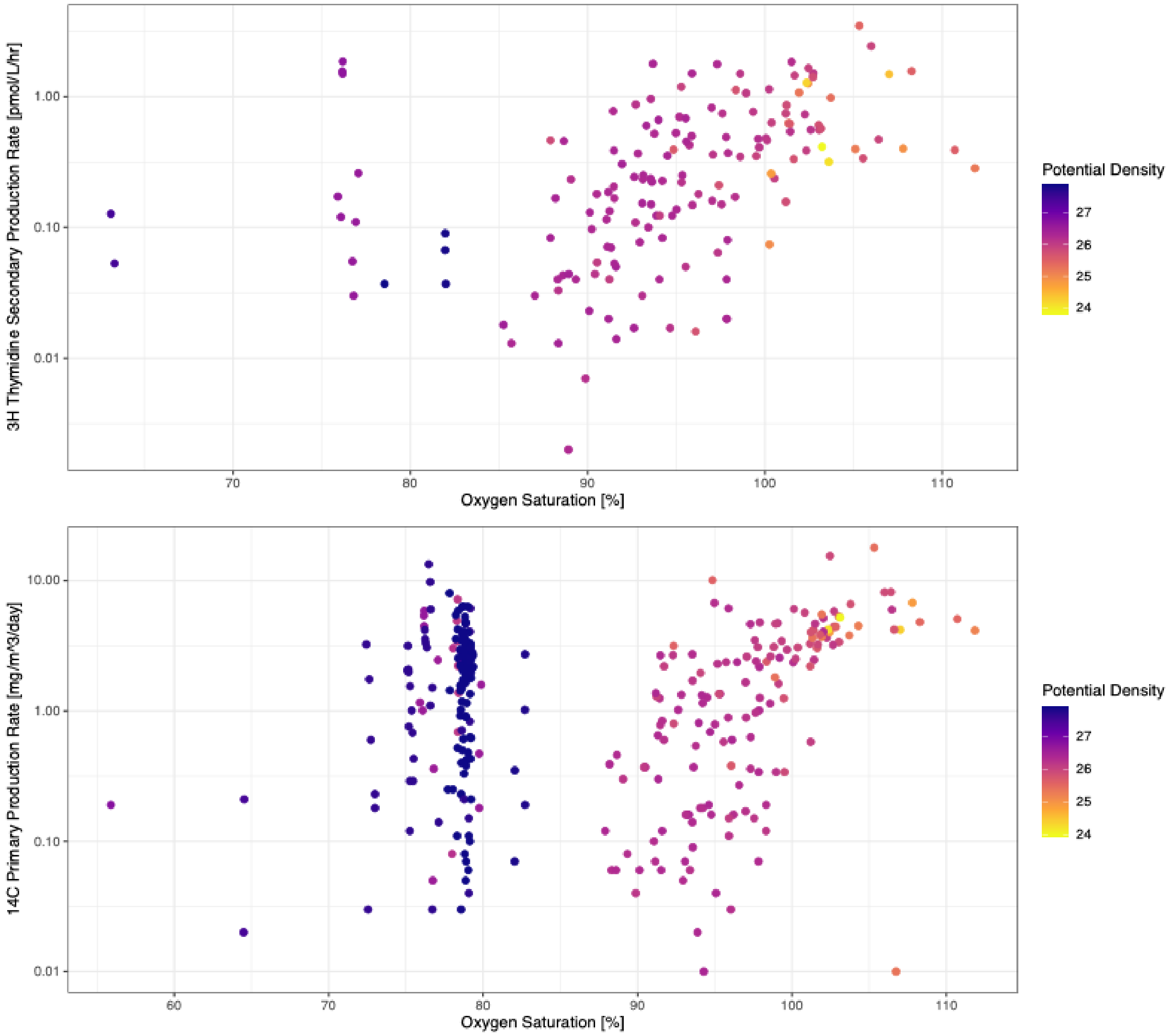

Supplementary Figure 2. Historical relationship between primary production, secondary production, and oxygen saturation in sub mixed-layer depths. Data shown are BATS *in situ* rate measurements taken between the surface and 200m depth in the stratified season (May-October). Points are colored by potential density calculated from BATS CTD profile data, and x-axis indicates the oxygen saturation at the experiment depth based on thermodynamic calculations described in *Methods*. Spearman rank correlations were calculated from experiments with oxygen saturation above 90% to avoid samples from deep mixing and subphotic depths (note that samples with low oxygen saturation in the figures have higher density, indicating intrusion of deeper water). Bacterial production measured through ^{3}H-labeled thymidine incorporation had a rank correlation of $\rho = 0.490, \mathrm{p} = 4 * 1e - 8$.

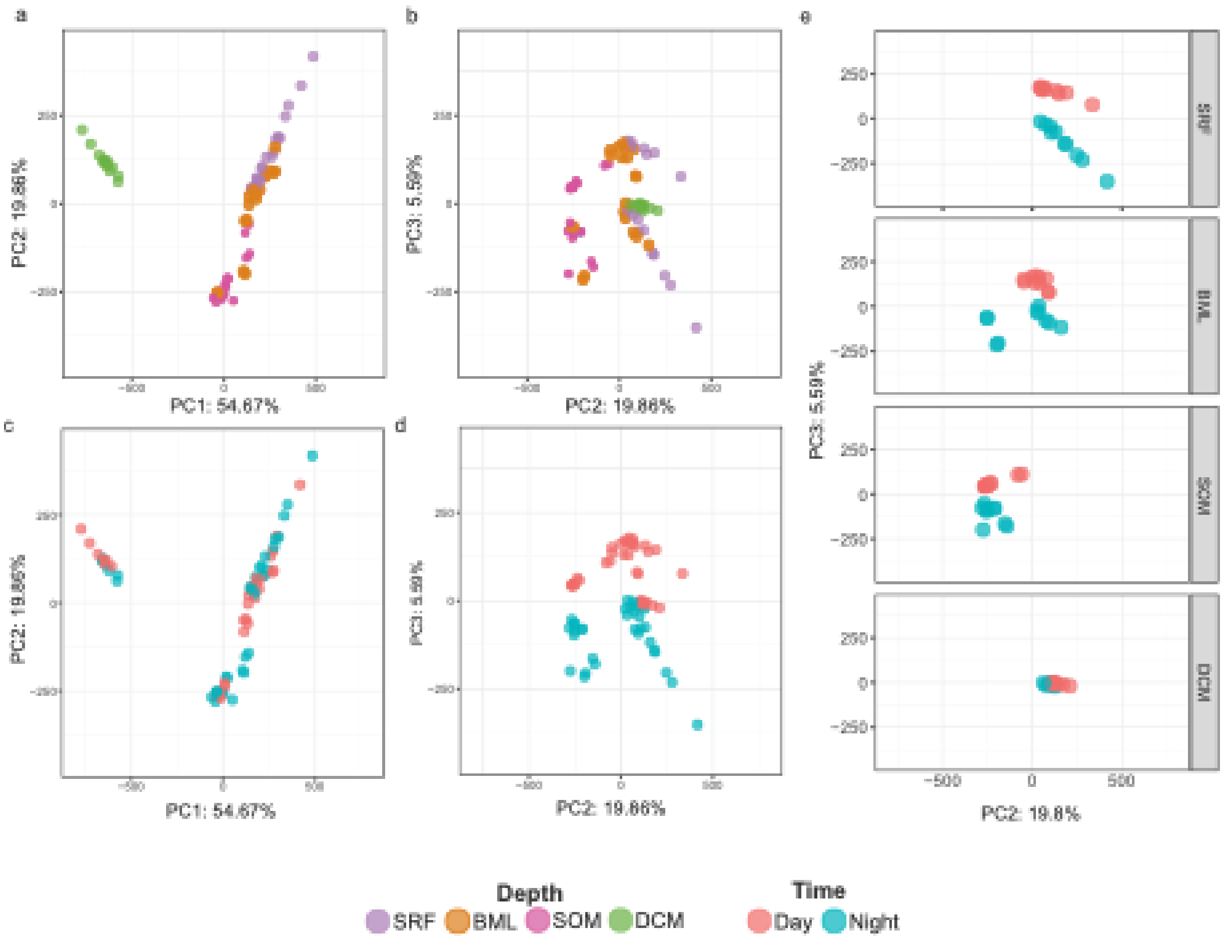

Supplementary Figure 3. Principal components (PC) analysis of community metatranscriptomes show shifts in expression profiles with depth and time. PCs were generated using normalized transcripts of all assembled genes across the co-assembly. a) PC1 against PC2 color coded by depth. b) PC2 against PC3 color coded by depth. c) PC1 against PC2 color coded by day (8:00, GMT-3) versus night (20:00, GMT-3). d) PC2 against PC3 color coded by day versus night. e) PC2 against PC3 color coded by time, plotting each depth separated into panels. Each dot is an independent biological replicate of the given depth and time. SRF = surface (day n=9, night n=10), BML = bottom of the mixed layer (day n=8, night n = 6), SOM = subsurface oxygen max (day n= 12, night n=13), DCM = deep chlorophyll max (day n= 8, night n=5)

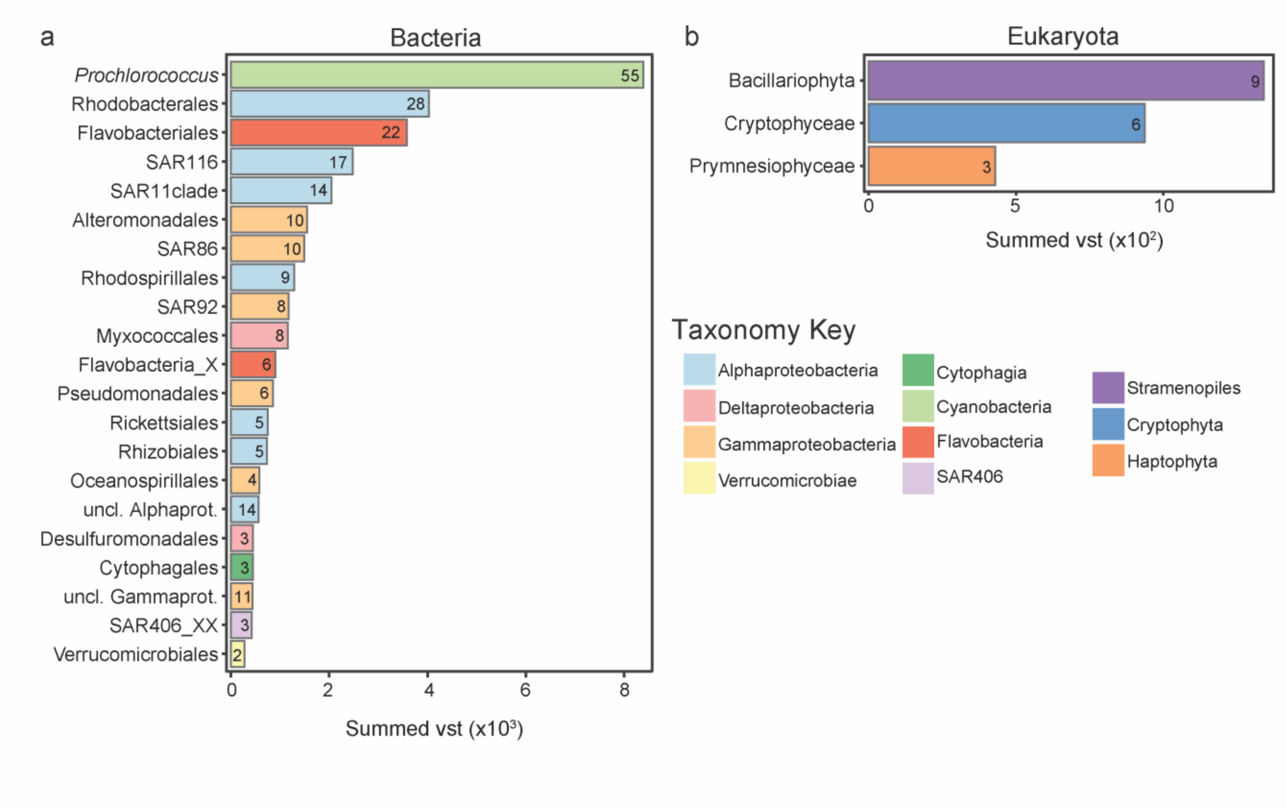

Supplementary Figure 4. Taxonomic annotations of *rpoB/RPB1* harbored on individual contigs (*rpoB/RPB1* hit) with significantly increased transcripts at the SOM. a) Summarized transcript abundances of top bacterial *rpoB/RPB1* hits with significantly elevated transcript abundances (Dunn test, BH-adjusted $p \leq 0.1$) specific to the SOM using order-level taxonomic annotation. *Prochlorococcus*-like *rpoB*s are annotated to the genus level. b) Summarized transcript abundances of top eukaryotic *RPB1* hits with significantly elevated transcript abundances (Dunn test, BH-adjusted $p \leq 0.1$) specific to the SOM using class-level taxonomic annotation. The number of individual *rpoB/RPB1* hits harbored on different contigs detected as significantly elevated at the SOM is shown within each bar, color coded by class-level information, for both panel a and b.

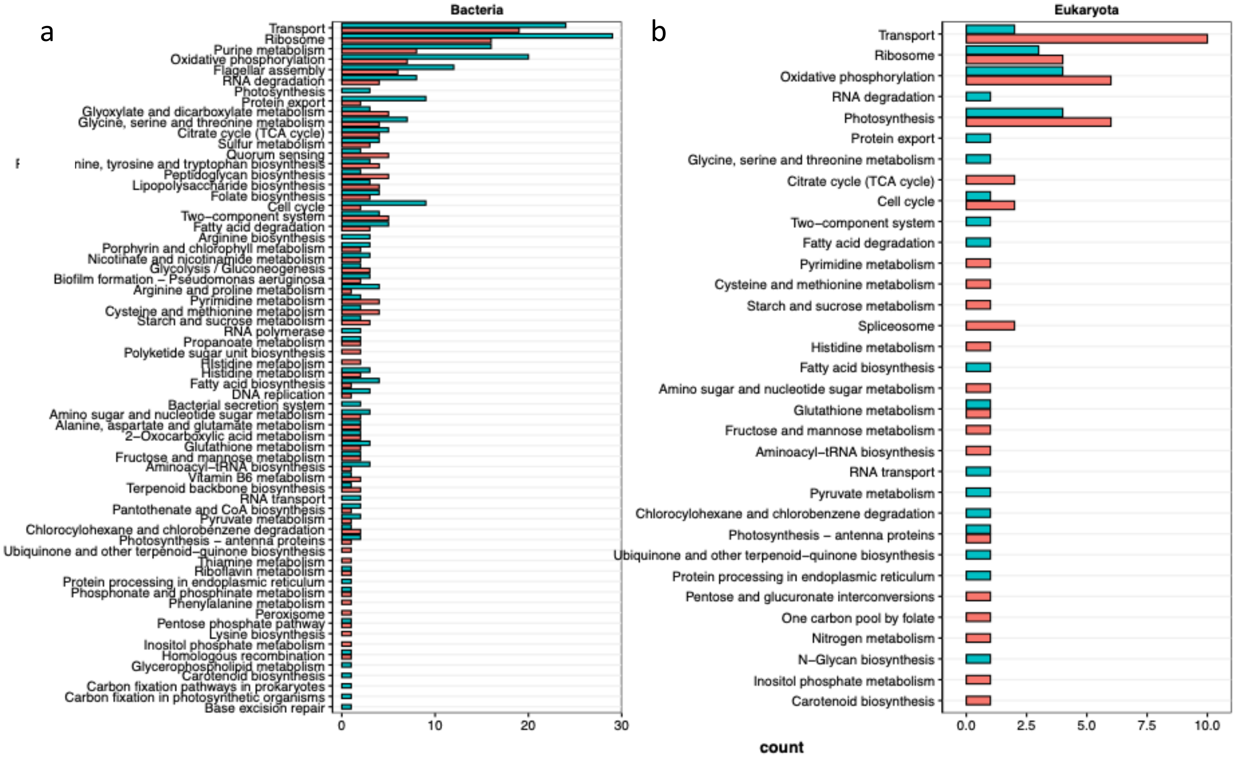

Supplementary Figure 5. Number of expressed KEGG pathways either uniquely heightened or lowered at the SOM relative to the surface, base of the mixed layer, and deep chlorophyll max. Top KEGG Orthology (KO) pathways assigned to KOs detected as significantly (Dunn-test, BH-adjusted p≤0.1) heightened or lowered at the SOM for a) Bacteria and b) Eukaryotes. Red = heightened at the SOM, Blue = lowered at the SOM.

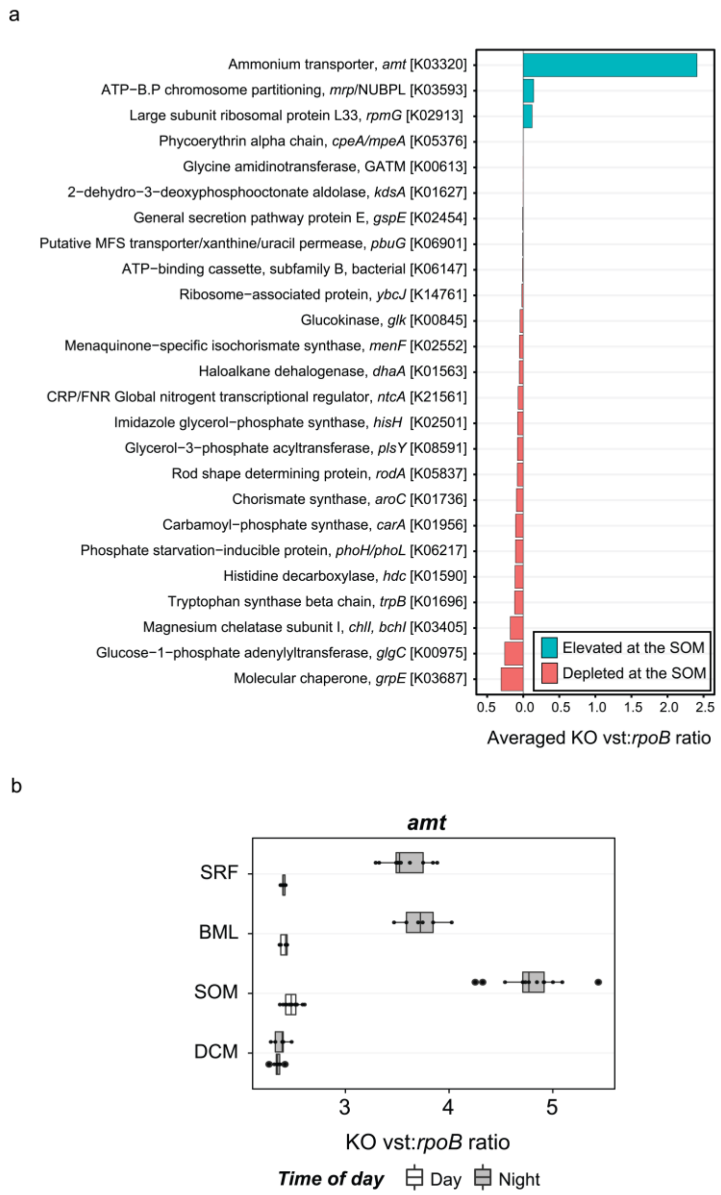

Supplementary Figure 6. *Prochlorococcus*-specific genes with either uniquely heightened or lowered transcript values at the SOM. a) KOs assigned to *Prochlorococcus* with significantly (Dunn-test, BH-adjusted $p \leq 0.1$) elevated or depleted transcript values at the subsurface oxygen maxima (SOM) relative to the surface (SRF), base of the mixed layer (BML) and deep chlorophyll maxima (DCM), displayed as the ratio of the KO VST to bulk *Prochlorococcus rpoB* VST (VST ratio) within each sample, averaged across the entire dataset. b) Depth and time-related trends of the VST ratio of the *amt* gene (ammonium transporter gene, K03320). Each dot is an independent biological replicate of the given depth and time. SRF = surface (day n=9, night n=10), BML = bottom of the mixed layer (day n=8, night n = 6), SOM = subsurface oxygen max (day n= 12, night n=13), DCM = deep chlorophyll max (day n= 8, night n=5). In the boxplot, the center line indicates the median, the box edges represent the 25th (lower, quartile 1) and 75th (upper, quartile 3) percentiles, and the whiskers extend to the smallest and largest values within 1.5 times the interquartile range below quartile 1 and above quartile 3, respectively.

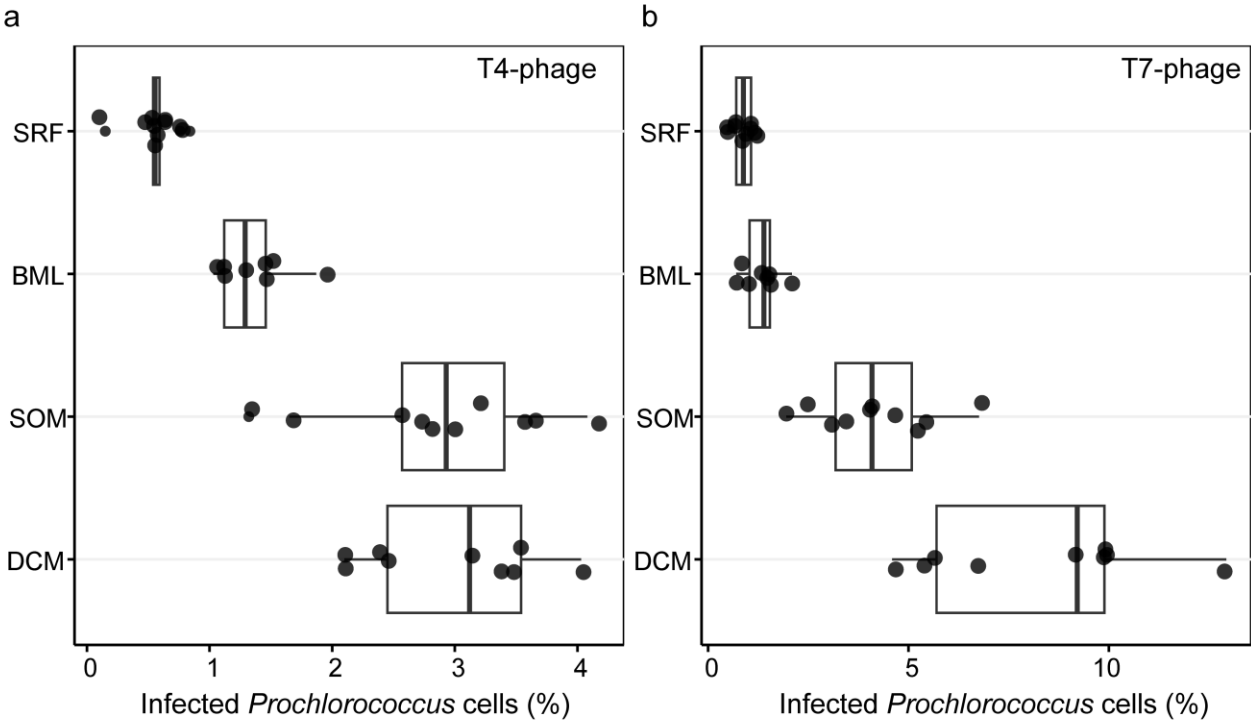

Supplementary Figure 7. **Extent of infection of *Prochlorococcus* cells by T4-like and T7-like cyanophages.** Percent infected *Prochlorococcus* cells by a) T4-like cyanophages and b) T7-like cyanophages. Infection was determined by the iPolony method quantifying the number of *Prochlorococcus* cells that have detected phage DNA, then calculating the percent of cells with phage DNA within the total number of cells interrogated. Each circle is an independent water sample (a biological replicate) collected at 8 am or 8 pm at each of the 4 depths across the cruise (SRF n=10, BML n=6, SOM n=10, DCM n=10). In the boxplots, the center line indicates the median, the box edges represent the 25th (lower, quartile 1) and 75th (upper, quartile 3) percentiles, and the whiskers extend to the smallest and largest values within 1.5 times the interquartile range below quartile 1 and above quartile 3, respectively.

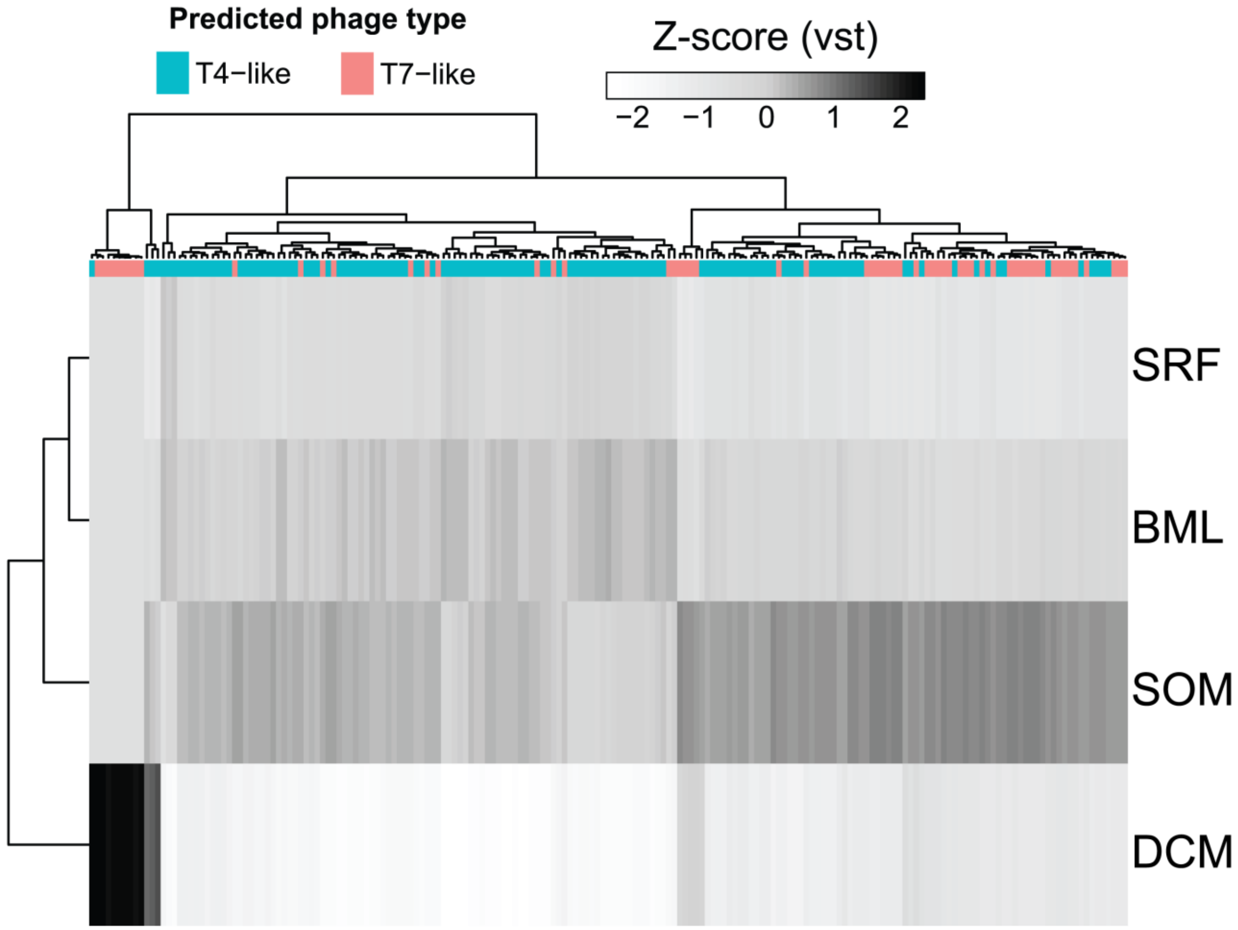

Supplementary Figure 8. Expression patterns of all potential T7-like and T4-like phages broadly infecting prokaryotes detected in the viromics dataset. Hierarchical clustering of normalized transcript abundance (VST) across entire vOTU scaffolds with significantly different transcript abundances as a function of depth. Values are averaged across depth for each vOTU. Putative taxonomy was determined by screening for T7-like and T4-like phages marker proteins on each scaffold.

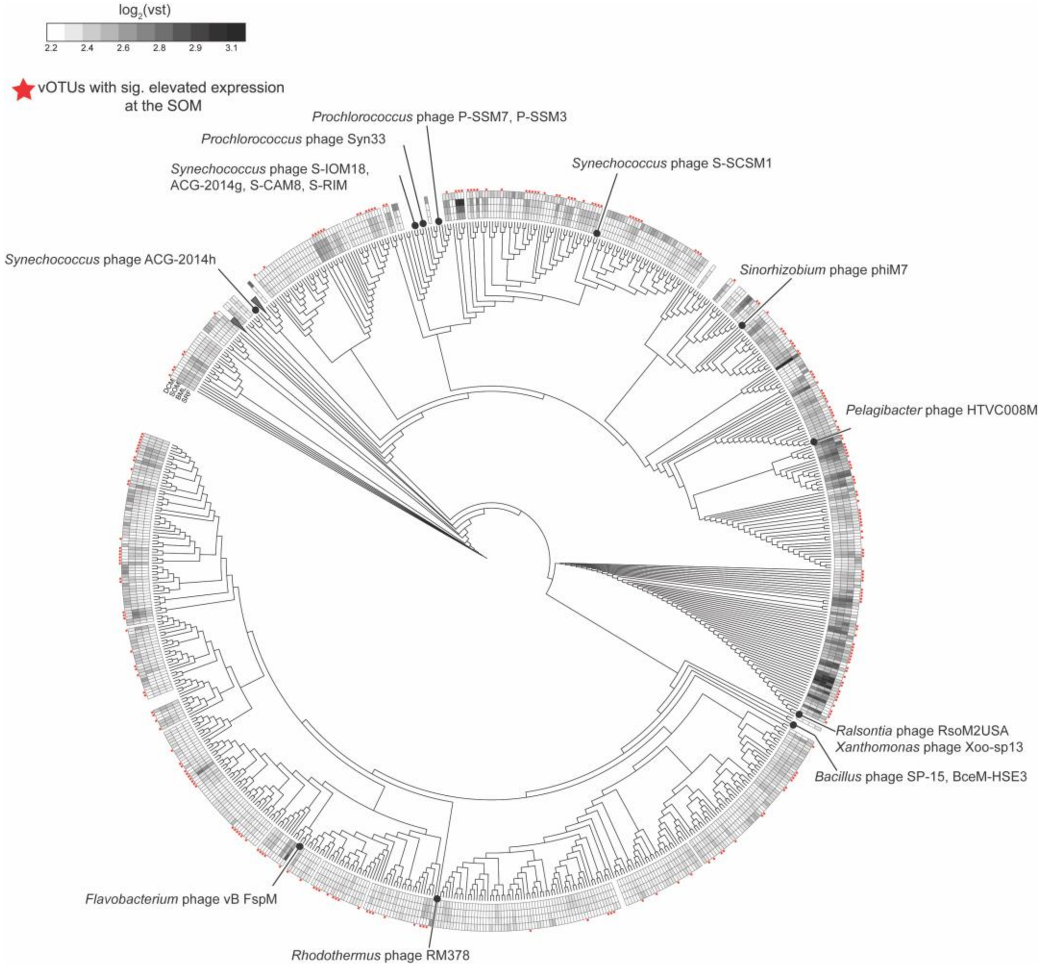

Supplementary Figure 9. Expression of T4-like vOTUs from the viromes broadly infecting prokaryotes. Phylogeny of T4-phage major capsid proteins (gp23) detected on vOTU scaffolds. Branches are color coded by their putative family-level assignment. The heatmap surrounding the tree shows depth-integrated $\log_2$(VST) values, from the SRF (inner ring) to the DCM (outer ring). vOTU gp23 transcripts with significantly elevated values at the SOM (Dunn-test, BH-adjusted $p < 0.1$) are indicated with a red star. References with assigned hosts used in the base tree are shown in their locations along the branches.

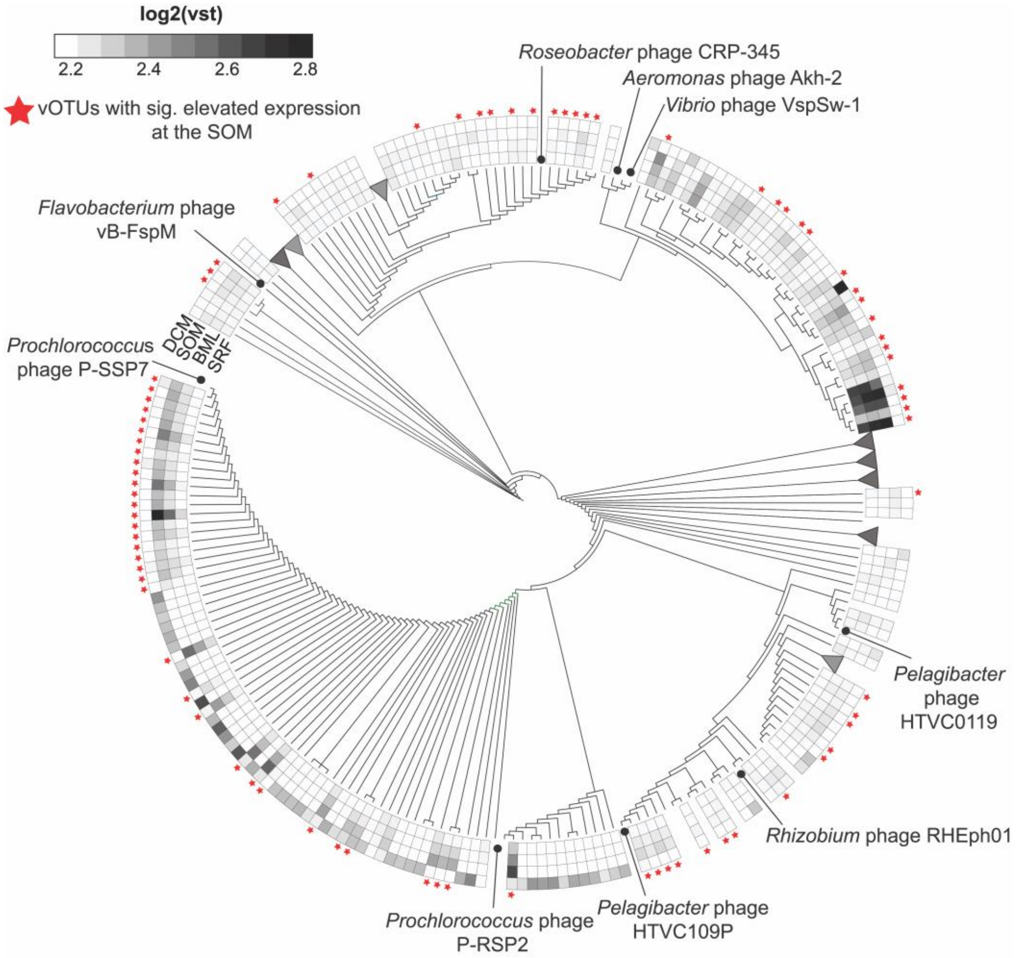

Supplementary Figure 10. Expression of T7-like vOTUs from the viromes broadly infecting prokaryotes. Phylogeny of DNA polymerase alpha-subunit (DNA Pol A) hallmark proteins detected on vOTU scaffolds. Branches are color coded by their putative family-level assignment. The heatmap surrounding the tree shows depth-integrated $\log_2$(VST) values, from the SRF (inner ring) to the DCM (outer ring). vOTU DNA Pol A transcripts with significantly (Dunn-test, BH-adjusted $p < 0.1$) elevated values at the SOM are indicated with a red star. References used in the base tree are shown in their locations along the branches.

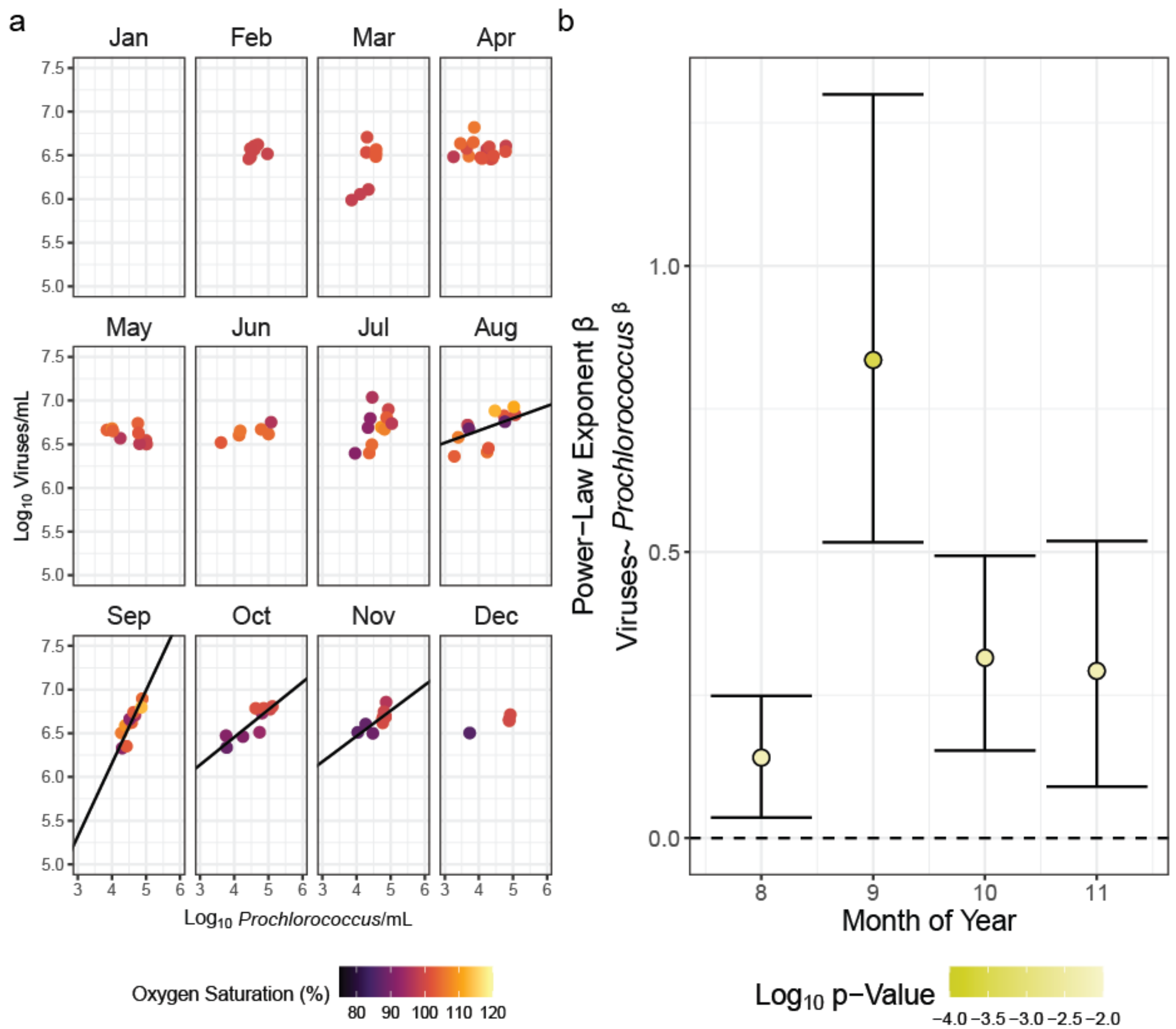

Supplementary Figure 11. Seasonal emergence of power-law relationships between *Prochlorococcus* abundance and bulk virus-like-particle (VLP) abundances across the BATS historical time series. a) Observations of VLP counts from Parsons *et al.*[3] with paired *Prochlorococcus* counts from the BATS time series, colored by oxygen saturation calculated from CTD oxygen, temperature, and salinity data. Solid line is major-axis regression best fit of the relationship between $\log_{10}$(*Prochlorococcus*) and $\log_{10}$(VLP). Major-axis regression lines are only plotted when the slope is significantly different from 0 based on bootstrapped simulations to estimate uncertainty in the slope. b) Power law exponents (linear regression of data plotted in panel a, which has log-log axes) by month in the form $V \sim cP^{\beta}$, where *V* is concentration of virus-like particles, *c* is a constant (intercept in panel a), *P* is the concentration of *Prochlorococcus* cells, and β is the exponent relating the relative increase in virus-like particles to an increase in *Prochlorococcus* concentration (the slope in panel a) . Points represent point estimates of the power law exponent (slope of the regression in panel a), and vertical bars represent 95% confidence intervals on the estimate of the slope. The model significance as indicated by fit p-value is the color of the point. Slopes with uncertainties are plotted for models with a significance level of $p<0.05$.

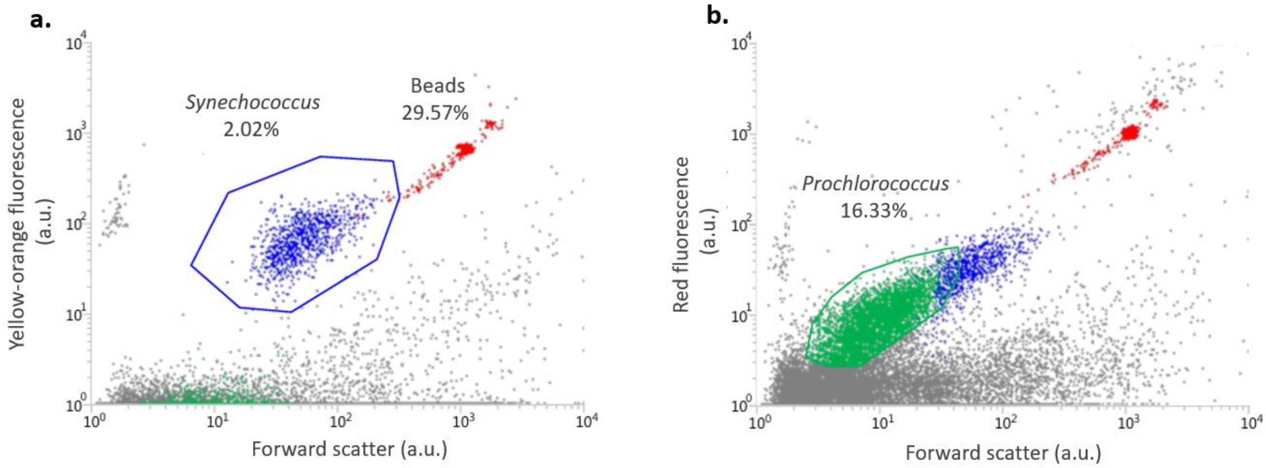

Supplementary Figure 12. Representative cytograms and gating of environmental community of picocyanobacterial. Populations are detected and gated based on forward scatter (a proxy for size) and autofluorescence of picocyanobacterial pigments. Hierarchical gating was employed gating first *Synechococcus* based on the yellow-orange fluorescence and phycoerythrin (a) and then *Prochlorococcus* based on the red fluorescence of chlorophyll *a* and lack of yellow orange fluorescence (b). Yellow-orange fluorescence was detected with emission at 580/30 nm and red fluorescence was detected with emission at 692/40 nm. Optical properties (fluorescence and scatter) are measured in arbitrary units (a.u.). The gating strategy in this panel was used to generate the cells/mL data reported in main Figure 5a. Heterotrophic bacteria cells/mL in main Figure 5b was calculated by subtracting the events colored in blue and green by the total events collected using SYBR Green staining (see Methods).
