# Supplementary material for: Seasonal enhancement of the viral shunt catalyzes a subsurface oxygen maximum in the Sargasso Sea
Source: Nat Commun. 2025 Dec 6;17:352. doi: 10.1038/s41467-025-67002-1 (PMC12796325; doi:10.1038/s41467-025-67002-1)
Supplement: Supplementary file 2 — Description of Additional Supplementary Files [file 41467_2025_67002_MOESM2_ESM.pdf]

Description of additional supplementary files

Supplementary Data 1. Flow cytometric and polony quantification of bacteria and phages. Calculation of ratios are also shown.

Supplementary Data 2. All detected KO-annotated genes with significantly (BH-adjusted p<=0.1) increased or decreased KO vst:*rpoB* ratios at the SOM relative to the surface (SRF), base of the mixed layer (BML), and deep chlorophyll max (DCM). The p-values were derived using the Kruskal-Wallis test followed by Dunn's multiple comparison testing using the Benjamini and Hochberg method.

Supplementary Data 3. Transporters with significantly (BH-adjusted p<0.1) elevated or depleted transcripts at the SOM relative to the Surface (SRF), base of the mixed layer (BML), and deep chlorophyll maxima (DCM) organized by taxon type (*Prochlorococcus*, heterotrophic bacteria, eukaryota) and transporter type. The p-values were derived using the Kruskal-Wallis test followed by Dunn's multiple comparison testing using the Benjamini and Hochberg method.

Supplementary Data 4. DESEQ2 results for all DNA Pol A and GP23 carrying vOTU scaffolds . P-values (padj) were obtained via DESEQ2 using the wald test and corrected for multiple testing using the Benjamini and Hochberg method

Supplementary Data 5. SRA id's for raw data included in the metatranscriptome and metavirome analysis
