# Supplementary material for: Seasonal enhancement of the viral shunt catalyzes a subsurface oxygen maximum in the Sargasso Sea
Source: Nat Commun. 2025 Dec 6;17:352. doi: 10.1038/s41467-025-67002-1 (PMC12796325; doi:10.1038/s41467-025-67002-1)
Supplement: Supplementary file 4 — Reporting Summary [file 41467_2025_67002_MOESM4_ESM.pdf]

nature research

Corresponding author(s): Steven Wilhelm, Joshua Weitz

Last updated by author(s): November 10 2025

Reporting Summary

Nature Research wishes to improve the reproducibility of the work that we publish. This form provides structure for consistency and transparency in reporting. For further information on Nature Research policies, see Authors & Referees and the Editorial Policy Checklist.

Statistics

For all statistical analyses, confirm that the following items are present in the figure legend, table legend, main text, or Methods section.

n/a	Confirmed	
☐	☒	The exact sample size (*n*) for each experimental group/condition, given as a discrete number and unit of measurement
☐	☒	A statement on whether measurements were taken from distinct samples or whether the same sample was measured repeatedly
☐	☒	The statistical test(s) used AND whether they are one- or two-sided<br>*Only common tests should be described solely by name; describe more complex techniques in the Methods section.*
☐	☒	A description of all covariates tested
☐	☒	A description of any assumptions or corrections, such as tests of normality and adjustment for multiple comparisons
☐	☒	A full description of the statistical parameters including central tendency (e.g. means) or other basic estimates (e.g. regression coefficient) AND variation (e.g. standard deviation) or associated estimates of uncertainty (e.g. confidence intervals)
☐	☒	For null hypothesis testing, the test statistic (e.g. *F*, *t*, *r*) with confidence intervals, effect sizes, degrees of freedom and *P* value noted<br>*Give P values as exact values whenever suitable.*
☒	☐	For Bayesian analysis, information on the choice of priors and Markov chain Monte Carlo settings
☒	☐	For hierarchical and complex designs, identification of the appropriate level for tests and full reporting of outcomes
☒	☐	Estimates of effect sizes (e.g. Cohen's *d*, Pearson's *r*), indicating how they were calculated

Our web collection on statistics for biologists contains articles on many of the points above.

Software and code

Policy information about availability of computer code

Data collection

No custom or commercial software was used to retrieve the BATS and CTD data

Data analysis

BATS historical CTD profiles were processed using the Gibbs Seawater Toolbox (https://www.teos-10.org/pubs/gsw/html/gsw_front_page.html) using the python 3.4.0 distribution through R via reticulate package v1/24
Diel periodicity analysis of CTD measurements was done using RAIN v.1.30.0 (Thaben & Westermark, 2014)
Type-II regression models were run using the R v4.2.1 package lmodel2 v.1.7-3
Metatranscriptomic libraries were filtered/trimmed using BBDuk v38.67 and BBMap v38.84 in the BBtools packages. Trimmed reads were assembled using MEGAHIT v1.2.9 and gene calls were done using MetaGeneMark v3.38. Reads were mapped to the assembly using BBMap v38.84 and tabulated using featureCounts from Subread package v2.0.1. Functional annotation was done using eggNOG-mapper v2.1.4 and EUKulele v2.1.0 for taxonomy (against the PhyloDB database). DESeq2 v.1.34.0 using R v4.1.3 was used for VST normalization of reads. Statistical analysis was done in R v4.1.3 using prcomp for principal components analysis (package stats v4.3.1). Kruskal-wallace tests were done using kruskal.test (package stats v4.3.1), dunn's multiple comparisons test using dunn.test v1.3.5 method = "bh".
For metaviromics, BBDuk v38.67, MEGAHIT v1.2.9, Virsorter 2 SOP 2.2.3, CheckV v0.8.1, Clustergenomes (https://github.com/simroux/ClusterGenomes), DRAM v1.4.0 were used. For viral hallmark protein phylogenetic trees, MEGA7 was used to run ClustalW, trimAl v1.2 was used to trim the alignment, and PhyML 3.0 was used to construct the maximum likelihood tree. ITOL v.4 was used to visualize the trees .
BD FACSDive v8 was used for flow cytometery and GraphPad Prism v 9.5.1 was used fro statistical analysis of the flow cytometry data.

Code used for analyses and generation of figures presented in this paper are available on github at the url https://github.com/d-muratore/virus_o2max. A release of this repository has been archived via zenodo (https://zenodo.org/records/17049625).

References Cited:

Thaben, P.F. & Westermark, P.O. Detecting rhythms in time series with RAIN. Journal of Biological Rhythms 29, 391-400 (2014)

Wilhelm, S.W., LeCleir, G.R., Sullivan, M.B. & Weitz, J.S. CTD Depth Profile Cast Data for the InVirT-2019-BATS (Bermuda Atlantic Time Series) project taken in the on board of the R/V Atlantic Explorer AE1926 in 2019. Biological and Chemical Oceanography Data Management Office (BCO-DMO). (2021).

For manuscripts utilizing custom algorithms or software that are central to the research but not yet described in published literature, software must be made available to editors/reviewers. We strongly encourage code deposition in a community repository (e.g. GitHub). See the Nature Research guidelines for submitting code & software for further information.

Data

Policy information about availability of data

All manuscripts must include a data availability statement. This statement should provide the following information, where applicable:

- Accession codes, unique identifiers, or web links for publicly available datasets
- A list of figures that have associated raw data
- A description of any restrictions on data availability

Raw metatranscriptomic data used to generate Figure 4, Figures S3-S6, and Figures S8-S10 is available through the JGI under project ID #505733. Raw viromic sequencing data used to generate Figures S8-S10 is available through the JGI under project ID #505733. Counts used for flow cytometry and Polony/iPolony assays used to generate Figures 5-6 , and Figure S7 are available in Supplementary Table 1. All AE1926 CTD cruise data used to generate Figure 1 and Figure S1 is available via BC-DMO (See Wilhelm et al. [ref 60]). Historical BATS data used to generate Figures 2-3, and Figure S11 are available on http://bats.bios.edu/bats-data/ . The historical BATS isotope incorporation experiment data used to generate Figure S2 is available on https://www.dropbox.com/scl/fo/x7xvlmyzqh9t9tfpe8ffg/AEBrkFFxVFvlO31MJmd9pcY?rlkey=7v73mfwhd78fdtxvfhk4v4kca&e=1&dl=0

All code and data used to generate the figures are available on github via https://github.com/d-muratore/virus_o2max and archived on zenodo (10.5281/zenodo.17049625). Intermediate data files and public datasets used to generate the figures are available on figshare via DOI:10.6084/m9.figshare.30047524. .

Field-specific reporting

Please select the one below that is the best fit for your research. If you are not sure, read the appropriate sections before making your selection.

☐ Life sciences ☐ Behavioural & social sciences ☒ Ecological, evolutionary & environmental sciences

For a reference copy of the document with all sections, see nature.com/documents/nr-reporting-summary-flat.pdf

Ecological, evolutionary & environmental sciences study design

All studies must disclose on these points even when the disclosure is negative.

Study description	The data presented in the study are taken from a 2019 Lagrangian cruse at the Bermuda Atlantic Time Series, where water samples from different depth strata were collected using a CTD Rosette equipped with 24x12 Niskin bottles. Water samples were taken every 12 hours at 4 distinct depths, or every 4 hours at surface depths, based on the CTD profiles at a given cast, over 6 days (October 12-17th 2019).
Research sample	The samples consist of a mixed community of naturally occurring eukaryotic & prokaryotic phytoplankton, heterotrophic bacteria , and viruses of differing proportions depending on the time and depth sampled. These samples were chosen as they are representative of the Sargasso Sea microbial community. The existing historical BATS data contains oceanographic and biological data collected monthly by the BATS Team.
Sampling strategy	Sampling size was determined based on availability of water at a given cast, which was influenced by the number of depths needed and volume of water per sample needed for each measurement taken. To increase diel- and depth-resolution, we set out to sample every 12 hours for ~6 days. The resultant sample size was dictated by success of sampling and sample processing .
Data collection	CTD data were automatically collected by AC, DM, and SW and which was manually with pen and paper at the time of sampling.
Timing and spatial scale	Diel samples (two surface depths sampled every 4 h, four depths sampled every 12 hours) were collected for 6 days (October 12-17th 2019). This scheme was chosen based on water availability and amount of time needed to process each sample.
Data exclusions	We did not include an analysis of the two surface depths sampled every 4 h (and instead, focused on depth profiles collected every 12 h) as this was used for a different study (diel surface cycling).
Reproducibility	Because this study was performed in the field and depends on the given conditions at a certain time, exact reproducibility of the results is not guaranteed. However, we note that the depth profile observed at the time of sampling occurs predictably each year based on historical BATS data, and so similar outcomes are likely.
Randomization	We utilized CTD depth data to assign each sample to a depth feature, and so the samples were not randomly assigned.
Blinding	Blinding is not relevant to this study as each sample was assigned a depth based on the CTD data (explained in the Methods).

Did the study involve field work? ☒ Yes ☐ No

Field work, collection and transport

Field conditions	Conditions were mainly calm and sunny throughout the cruise, with air temperature ranging from 25.3 - 26.4 degrees C, wind speed ranging from 1.8-8.3 kts, and humidity around 67-76%.
Location	Sampling started at the Bermuda Atlantic Time Series (31.5 N, 64.2 W) and ended around 31.8 N, 64.4 W. Water samples were collected from the surface (~5 m depth) to the deep chlorophyll maximum (~120 m depth).
Access and import/export	An application to undertake Scientific Research Activities was submitted and approved by the Government of Bermuda, Department of Environmental and Natural Resources prior to the cruise. An export permit was obtained >2 weeks prior to the cruise as well.
Disturbance	No disturbance reported.

Reporting for specific materials, systems and methods

We require information from authors about some types of materials, experimental systems and methods used in many studies. Here, indicate whether each material, system or method listed is relevant to your study. If you are not sure if a list item applies to your research, read the appropriate section before selecting a response.

Materials & experimental systems

n/a	Involved in the study
☒	☐ Antibodies
☒	☐ Eukaryotic cell lines
☒	☐ Palaeontology
☒	☐ Animals and other organisms
☒	☐ Human research participants
☒	☐ Clinical data

Methods

n/a	Involved in the study
☒	☐ ChIP-seq
☐	☒ Flow cytometry
☒	☐ MRI-based neuroimaging

Flow Cytometry

Plots

Confirm that:

☒ The axis labels state the marker and fluorochrome used (e.g. CD4-FITC).

☒ The axis scales are clearly visible. Include numbers along axes only for bottom left plot of group (a 'group' is an analysis of identical markers).

☒ All plots are contour plots with outliers or pseudocolor plots.

☒ A numerical value for number of cells or percentage (with statistics) is provided.

Methodology

Sample preparation	Samples were collected from the CTD Niskin bottle, prefiltered through a 20 um mesh and fixed in 0.125% glutaraldehyde, flash frozen and stored at -80C as outlined in the Methods.
Instrument	Influx BD flow cytometer equipped with a 488 nm and a 457 nm laser and a 70 µm nozzle tip (BD Biosciences)
Software	BD FACSDiva™
Cell population abundance	Prochlorococcus and Synechococcus cells were sorted at concentrations between 800-1000 cells/uL. Cell abundance was determined by measuring the volume of the sample analyzed .
Gating strategy	Total bacterial cells stained with SYBR Green I were gated using green fluorescence exited with the 488 nm laser . Cyanobacteria (Prochlorococcus and Synechococcus) were gated based on autofluorescence and FSC. Prochlorococcus was detected by red fluorescence of chlorophyll a (emission at 692/640 nm) while Synechococcus was detected by orange fluorescence of phycoerythrin (emission at 580/30 nm). Heterotrophic bacteria were enumerated using SYBR Green and picocyanobacterial abundance was subtracted from SYBR Green stained cells to get heterotrophic bacteria cell counts.

☒ Tick this box to confirm that a figure exemplifying the gating strategy is provided in the Supplementary Information.
